# Carbon Modification of K_1.6_Fe_1.6_Ti_6.4_O_16_ Nanoparticles to Optimize the Dielectric Properties of PTFE-Based Composites

**DOI:** 10.3390/polym14194010

**Published:** 2022-09-25

**Authors:** Alexey Tsyganov, Maria Vikulova, Denis Artyukhov, Alexey Bainyashev, Vladimir Goffman, Alexander Gorokhovsky, Nikolay Gorshkov

**Affiliations:** 1 Department of Chemistry and Technology of Materials, Yuri Gagarin State Technical University of Saratov, 77 Polytecnicheskaya Street, Saratov 410054, Russia; 2 Department of Power and Electrical Engineering, Yuri Gagarin State Technical University of Saratov, 77 Polytecnicheskaya Street, Saratov 410054, Russia

**Keywords:** hollandite, sol–gel, carbon black, polymer composite, permittivity, dielectric loss

## Abstract

In this work, polymer matrix composites with the compositions PTFE/KFTO(H) and PTFE/KFTO(H)@CB and with filler volume fractions of 2.5, 5.0, 7.5, 15, and 30% (without and with carbon modification at a content of 2.5 wt.% regarding ceramic material) were produced by calendering and hot pressing and studied using FTIR, SEM, and impedance spectroscopy methods. Ceramic filler (KFTO(H)) was synthesized using the sol–gel Pechini method. Its structure was investigated and confirmed by the XRD method with following Rietveld refinement. The carbon black (CB) modification of KFTO(H) was carried out through the calcination of a mixture of ceramic and carbon materials in an argon atmosphere. Afterwards, composites producing all the components’ structures weren’t destroyed according to the FTIR results. The effect of carbon additive at a content of 2.5 wt.% relating to ceramic filler in the system of polymer matrix composites was shown, with permittivity increasing up to ε’ = 28 with a simultaneous decrease in dielectric loss (tanδ < 0.1) at f = 10^3^ Hz for composites of PTFE/KFTO(H)@CB (30 vol.%).

## 1. Introduction

Dielectrics based on polymer matrix composites have great potential for applications in various types of electronic components. This is mainly due to the properties of the polymer matrix, such as flexibility, chemical stability, and the ease and wide possibilities of molding products for various applications [1,2,3]. For example, they can be used as an insulating layer in an electric cable or as a film capacitor for energy storage [4,5,6,7,8]. However, polymer matrices have low values of permittivity, which limits their application in modern electronics [9]. In this regard, two- and three-phase composites based on different polymer matrices and various fillers, which are dielectrics or conductors, have been widely investigated. Effective composites with dielectric filler are usually complex oxide particles with a high permittivity dispersed in a polymer matrix [10]. Metal nanoparticles and various modifications of carbon nanomaterials are used as conductive fillers [10,11]. The most common ceramic fillers are usually materials with perovskite structure such as barium titanate and strontium titanate [12,13], as well as copper calcium titanate (CCTO) with a cubic structure and very high permittivity values [14,15,16,17]. The addition of only ceramic or conductive fillers to the polymer can improve the dielectric properties of the resulting composites, but there are limitations to their use. When using ceramic fillers, high permittivity values can only be achieved with high volume fractions of them, though at the same time this leads to a strong deterioration in the mechanical properties of the composites. With conductive fillers, a significant increase in permittivity above the percolation threshold can be achieved, which at the same time leads to a steep rise in dielectric losses due to their high electrical conductivity. Due to the disadvantages of ceramic and conductive fillers, many studies have focused on the study of three-phase composites based on co-added dielectric and conductive fillers in polymer matrices [18,19,20,21,22,23]. This approach contributes to a more efficient increase in permittivity and a decrease in the dielectric loss tangent and has been considered to be a potential solution for the next generation of dielectrics for personal electronics.

Potassium titanates with a hollandite structure doped with various transition metals can be an analogue of modern high-K materials as effective ceramic fillers for polymer matrices [24,25,26,27]. In several studies [28,29,30], the possibility of increasing the permittivity with the introduction of hollandite-like potassium titanates doped with iron, copper, and nickel ions into polymer matrices based on PTFE, PMMA, and epoxy has been shown. However, in these works, the titanate powders were produced by solid-state reaction and had particle sizes of a few micrometres, which can significantly reduce the effectiveness of the increase in permittivity. Additionally, the introduction of nanosized ceramic particles produced using the sol–gel method may be more effective. In addition, the use of hollandite-like powders in combination with carbon nanotubes as fillers for polymer matrices to improve their dielectric properties has been shown to be effective in prior research [30].

In this work, polytetrafluoroethylene (PTFE) was chosen as the polymer matrix because of its properties of flexibility, strength, chemical resistance, and ease of fabrication. As a ceramic filler, nanodimensional particles of potassium titanate K_1.6_Fe_1.6_Ti_6.4_O_16_ (KFTO(H)) with a hollandite structure due to its high permittivity were obtained. Carbon black (CB) was chosen as the conductive filler because of its availability, low cost, and high conductivity. The aim of this work was to investigate the dielectric properties of three-phase PTFE/KFTO(H)@CB composites in a wide frequency range.

## 2. Materials and Methods

### 2.1. KFTO Synthesis

K_1.6_Fe_1.6_Ti_6.4_O_16_ powder was synthesized using the Pechini sol–gel method. The raw materials used for the synthesis of nanopowders in the K_2_O-TiO_2_-Fe_2_O_3_ system were C_2_H_6_O_2_ (98.5%, Russian Standard 19710-2019, Aricon, Moskow, Russia), C_6_H_8_O_7_ (99.5%, Russian Standard 3652-69, Aricon, Moskow, Russia), KNO_3_ (98%, Russian Standard 4217-77, OAO “BUYSKIY HIMICHESKIY ZAVOD”, Buy, Russia), Fe(NO_3_)_3_ 9H_2_O (98%, TC 6-09-02-553-96, OAO “BUYSKIY HIMICHESKIY ZAVOD”, Buy, Russia), C_16_H_36_O_4_Ti (99%, Acros Organics, Geel, Belgium), HNO_3_ (65%, Russian Standard 4461-77, OAO “BUYSKIY HIMICHESKIY ZAVOD”, Buy, Russia), and NH_4_OH (25%, Russian Standard 3760-79, Aricon, Moskow, Russia).

**Figure 1:**
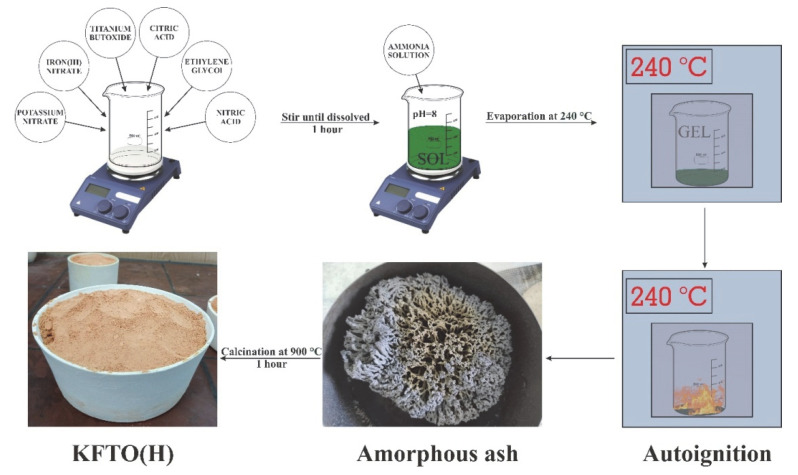
KFTO(H) synthesis scheme.

The nanopowder-producing method consisted of several steps. KNO_3_ and Fe(NO_3_)_3_ 9H_2_O were dissolved in a minimal amount of distilled water and then added to C_16_H_36_O_4_Ti in stoichiometric ratios corresponding to K_1.6_Fe_1.6_Ti_6.4_O_16_ (KFTO(H)). Aqueous solutions of HNO_3_, C_2_H_6_O_2_ as a metal ion chelating agent, and C_6_H_8_O_7_ as a crosslinking agent were added to the resulting mixture. Optimal stoichiometric ratios were experimentally found to be Ti:C_6_H_8_O_7_ = 1.5; Ti:C_2_H_6_O_2_ = 5.5; Ti:NO_3_ = 1.8. After the complete dissolution of C_16_H_36_O_4_Ti, 10% NH_4_OH solution was added to the solution until it reached pH = 8, following the formation of a sol. The next step, conducted according to the Pechini method, was to evaporate the solvent and form the polymer resin in a drying oven at 240 °C to initiate a self-sustaining combustion reaction and ensure the extraction of the NO_2_ released. The gel was melted and boiled with the formation of black foam and subsequently auto-ignited black ash. The combustion was continued for several minutes with a smoldering flame. The amorphous ash was crushed and calcined at 900 °C for 1 h. The resulting product was a highly dispersed KFTO(H) powder used for the preparation of PTFE/KFTO(H) composites. The synthesis scheme is shown in Figure 1.

### 2.2. PTFE/KFTO(H) Composites Producing

To prepare PTFE/KFTO(H)@CB composites, the resulting KFTO(H) powder was mixed with carbon black (Printex RX2B) (CB content in the mixture 2.5 wt.%) in a vibrating mill and then calcined in a muffle furnace at 600 °C in an argon atmosphere. This approach promotes the formation of a carbon shell on the surface of the ceramic particles [31]. The powders obtained and an aqueous dispersion of polytetrafluoroethylene (PTFE) (60 wt.%, TE-3865C, Dupont, Wilmington, DE, USA) were used to prepare the composites.

PTFE/KFTO(H) and PTFE/KFTO(H)@CB composites were produced by calendaring and the hot pressing method. KFTO(H) and KFTO(H)@CB powders (2.5 vol.%, 5 vol.%, 7.5 vol.%, 15 vol.%, 30 vol.%) were added to the PTFE aqueous dispersion (ρ = 3.44 g/cm^3^). Then, the dispersions were subjected to ultrasonic stirring, followed by washing with ethanol to remove surfactants and drying at 150 °C. The dried mixtures were placed in a cylindrical mold with a diameter of 12 mm and subjected to hot pressing at 360 °C at a pressure of 10 MPa for 1 h. As a result, composites were obtained in the form of discs with a thickness of ~1.5 mm. Both sides of the discs were covered by silver-containing adhesive (trademark Contactol K13, OOO Adecvat, Moscow, Russia) and dried at 120 °C to obtain the electrodes for use in the impedance measurements.

### 2.3. Characteristics Methods

The Thermo Scientific ARL X’TRA device (Ecublens, Switzerland) using Cu Kα radiation (λ = 0.15412 nm) was used to record the diffractogram of the synthesized ceramic filler. An Analysette-22 NanoTech laser particle size analyzer (Idar-Oberstein, Fritsch GmbH) was used to determine the particle size distribution of ceramic filler in the dispersion. The analysis of the powder morphology and composite samples was carried out using scanning electron microscopy (Aspex EXplorer, Aspex LLC, Framingham, MA, USA). FTIR spectra were obtained by an FT-801 spectrometer (Simex, Novosibirsk, Russia) using the ATR-FTIR method and analytical equipment single-reflection ATR. An Impedance Analyzer Novocontrol Alpha AN, (Novocontrol Technologies GmbH & Co. KG, Montabaur, Germany) was used to measure the dielectric properties of the composites via impedance spectroscopy. The frequency ranged from 0.01 Hz to 1.00 MHz with a voltage amplitude of 100 mV. The permittivity (ε′, ε″, and ε), conductivity (σ), and dielectric losses (tan δ) were found from the experimental values of the real and imaginary parts of the impedance (Z′ and Z″) using the standard computing operations.

## 3. Results and Discussion

**Figure 2:**
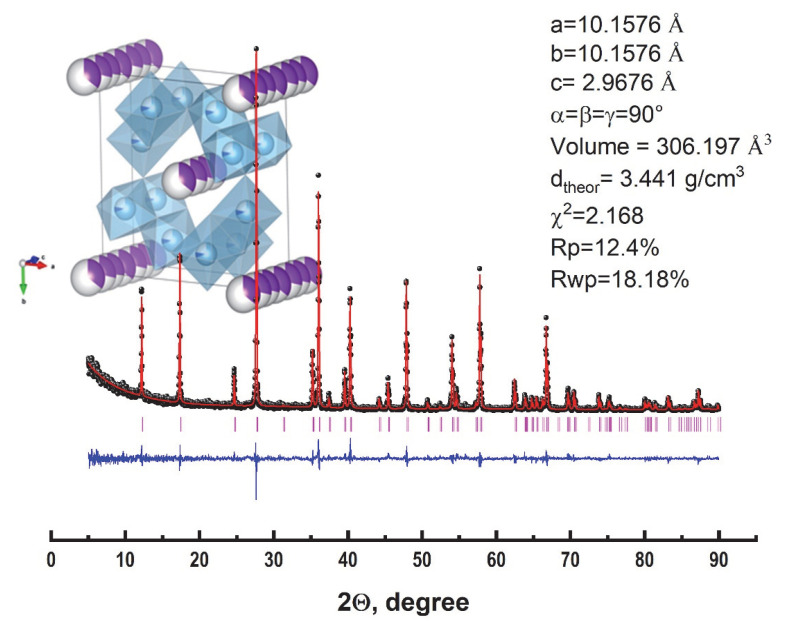
Combined X-ray diffraction patterns and Rietveld refinement of KFTO(H): black circles—experimental data, red line—calculated line, blue line—difference, purple vertical lines—Bragg positions.

The diffractogram of synthesized KFTO(H) powder and the Rietveld structural refinements performed using the software general structure analysis system (spatial group l4/m, JCPDS No. 77-0990) is shown in Figure 2. As can be seen, only narrow, high-intensity diffraction reflexes attributed to potassium titanate with a hollandite structure can be observed in the diffractogram, suggesting that the product is a monophase KFTO(H) powder with a good crystallinity after annealing at 900 °C. The plot of the final Rietveld refinement shown in Figure 2 features a good agreement between the experimental and calculated intensities in the tetragonal system with the spatial group I4/m. The calculated cell parameters were a = 10.1576 Å and c = 2.9676 Å and were similar to the compositions, given the ionic radii of the alloying metals used in research [32]. From the Rietveld refinement, the theoretical density was determined and used to prepare the composites and calculate their porosity.

**Figure 3:**
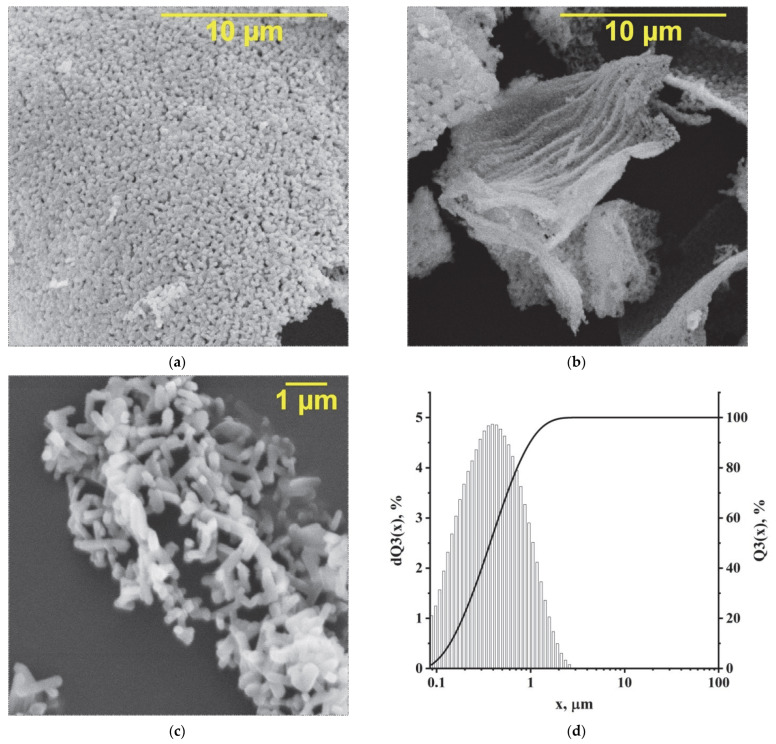
SEM microphotographs (**a**–**c**) and particle size distributions (**d**) of KFTO(H) nanopowder (x is particle size, μm; dQ3(x) is the differential percentage of particle volume entering the range between the minimum and maximum size; Q3(x) is the integral percentage of the particle volume relevant to the range in question, showing which fraction of the particle volume is below the specified size).

SEM microphotographs (a, b, c) and particle size distributions (d) of KFTO(H) powder are shown in Figure 3. The distribution is represented by particles with an average size of d_50_ = 400 nm which form agglomerates. The size and shape of agglomerates can varied, as seen from the electron microphotographs (Figure 3a,b). Single particles have a morphology similar to that seen in the image in Figure 3c. All crystals are needle-shaped, with square bases and flat faces, indicating a tetragonal structure. It should be noted that in the case of the use of the laser diffraction method, the conversion of the diffraction angle to the particle size is based on the equivalent sphere model.

**Figure 4:**
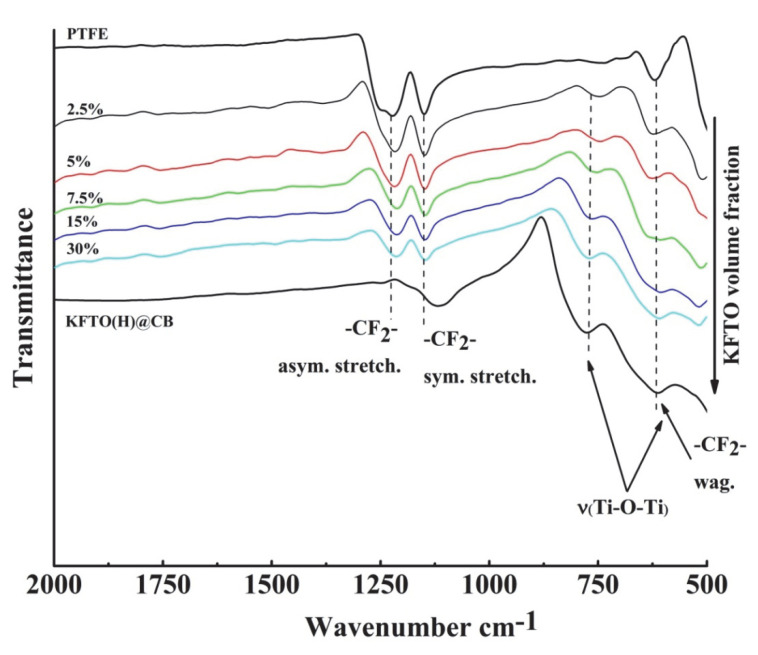
FTIR spectra of PTFE, KFTO(H)@CB, and polymer matrix composites based on them.

The study of the characteristic functional groups of the composite components as well as the confirmation of the preservation of the polymer and filler initial structures at their mixing was carried out using the FTIR spectroscopy method (Figure 4). Absorption bands at 1150 and 1220 cm^−1^, corresponding to symmetric and asymmetric stretching -CF_2_- vibrations, respectively, are clearly distinguishable in the FTIR spectrum of PTFE. Additionally, a lower-intensity absorption band at 615 cm^−1^, responsible for the wagging oscillation of similar polymer groups, can be observed [33]. The KFTO(H) filler was predominantly characterized by metal–oxygen chemical bonds, which appear on the FTIR spectrum as absorption bands associated with the valence vibrations of Ti-O-Ti bonds at 770 and 620 cm^−1^. The pre-application of the carbon modification of the ceramic filler to obtain the sample KFTO(H)@CB does not lead to significant changes in the structure and, consequently, FTIR spectrum. Regardless of the filler content, the FTIR spectra of the obtained composites show absorption bands for both the polymer matrix and the composite filler. This indicates the absence of chemical interaction between the composite components and catalytic processes characteristic of both oxide and carbon materials that lead to the degradation of PTFE, as well as changes in the KFTO(H)@CB structure.

**Figure 5:**
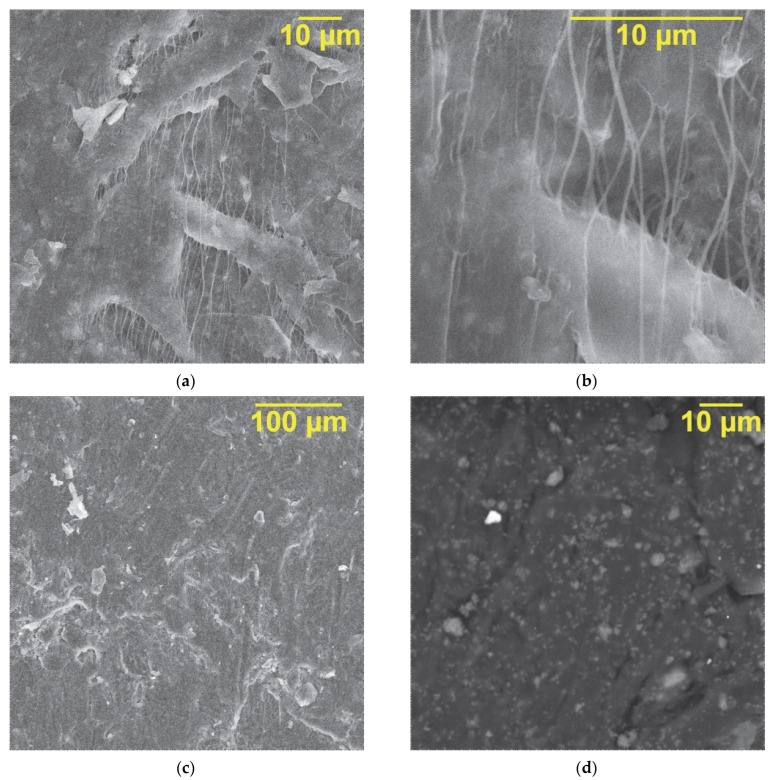
SEM images of PTFE/KFTO(H)@CB composites with 30 vol.% filler: (**a**,**b**) cross-section; (**c**) surface using primary electrons and (**d**) secondary electrons.

Scanning electron microscopy (SEM) images of a fractured cross-section of PTFE/KFTO(H)@CB composites with 30 vol.% ceramic filler in the polymer matrix are shown in Figure 5a,b. KFTO(H)@CB filler particles with dimensions less than 1 μm are uniformly surrounded by the PTFE polymer matrix. In addition, no agglomerated ceramic filler particles can be observed in the cross section of the composite. SEM images obtained of primary (Figure 5c) and secondary electrons (Figure 5d) also indicate the absence of agglomerates and a uniform distribution of the filler in the polymer matrix.

Porosity is an important parameter for the properties of composites, especially dielectric composites. The porosity can be determined using the following equation:

(1) $$V_{A}=\left(1-\frac{\rho_{A}}{\rho_{t}}\right)\cdot 100\%$$

(2) $$\rho_{t}=V_{f}\cdot \rho_{f}+V_{m}\cdot \rho_{m}=\frac{w_{f}+w_{m}}{w_{f}/\rho_{f}+w_{m}/\rho_{m}}$$

where $V_{A}$ is the composites’ porosity, %; $\rho_{t}$ is the theoretical density of composites, g/cm^3^; and $\rho_{A}$, $\rho_{f},$ and $\rho_{m}$ are the experimental density of the composites, filler, and polymer matrix, respectively, in g/cm^3^.

**Figure 6:**
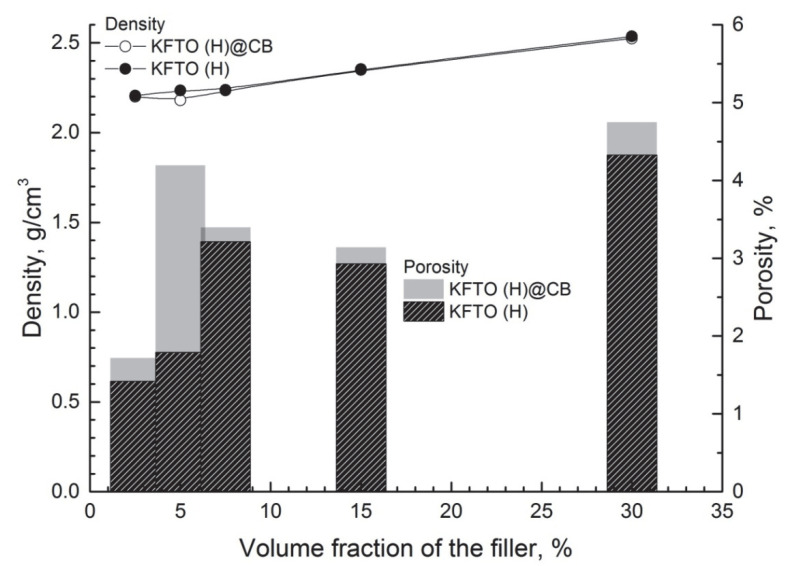
Porosity diagram of PTFE/KFTO(H)@CB and PTFE/KFTO(H) composites as a function of filler volume fraction.

The experimental density of the composites was measured using Archimedes’ method. The theoretical density was calculated considering that the densities of KFTO(H) and PTFE were 3.44 and 2.2 g/cm^3^, respectively. The porosity of the composites is shown in Figure 6. The porosity was about 1.5% at minimum KFTO(H) filler content and increased to 4.5% at 30 vol.% filler content. These porosity values were less than 5% and acceptable for composites of this type, indicating the minimal aggregation of filler particles [34]. It is worth noting that the modification of KFTO(H) particles with carbon leads to an increase in porosity at all filler volume fractions. This may be due to the fact that the particles of the original ceramic filler KFTO(H) by nature have a good adhesive contact with the polymer matrix, which deteriorates when their surface is modified with carbon. In conjunction with this, the high viscosity and low melting fluidity of PTFE result in the increased porosity of the composites.

**Figure 7:**
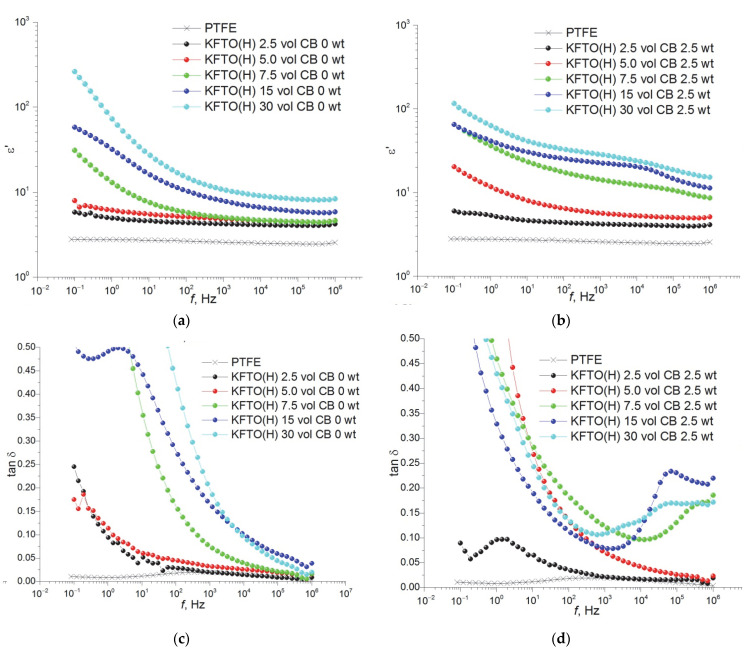
Dielectric properties of the frequency dependence of PTFE/KFTO(H) (**a**,**c**) and PTFE/KFTO(H)@CB (**b**,**d**) composites.

Figure 7 shows the frequency dependencies of permittivity (a, b) and dielectric loss tangent (c, d) for PTFE/KFTO(H)@CB and PTFE/KCTO(H) composites as a function of the filler volume fraction KCTO(H) at room temperature. The sample of composite with 2.5 vol.% of KFTO(H) has a minimal value of permittivity (ε′ = 4.2); at the same time, the composite containing 30 vol.% of ceramic filler shows the highest permittivity value of 8.3, while other samples have intermediate values at *f* = 1 MHz (Figure 7a). Similar to composites based on other fillers, the resulting permittivity values of the composites are much lower than those of KFTO(H)-based ceramics. This indicates that the polymer matrix largely determines the permittivity of the composite based on it. In addition, increasing the amount of filler causes interfacial polarization due to an increase in the connection of the PTFE–KFTO(H) interface, which in turn contributes to an increase in permittivity and dielectric losses. For all samples in the low-frequency region, a sharp increase in permittivity is observed, associated with the formation of interphase boundaries and the accumulation of charge carriers on these boundaries, which form dipoles under the influence of an external electric field. It should be noted that the use of small KFTO(H) particles contributes to a more effective increase in permittivity with decreasing porosity compared to larger particles of similar composition studied in [28]. This also indicates an increase in the number of interfacial boundaries and an important role for interfacial polarization in the mechanism of permittivity increasing.

As can be seen from the frequency dependence of the permittivity of PTFE/KFTO(H)@CB composites containing carbon filler, the permittivity increases significantly over the entire frequency range (Figure 7b). This increase is more noticeable in the high-frequency range; as a result, the permittivity becomes less frequency-dependent. The increase in permittivity is greater at higher concentrations of ceramic filler, as the amount of carbon in the composites also increases. The additive of carbon in the composites has a low influence on the permittivity values at low frequencies. This may be due to the fact that the carbon introduced into the composites completely covers the KFTO(H) particles, which results in there being no increase in the number of filler-polymer interfaces and causes the interfacial polarization to remain at the same level. It is known that the additive of conductive carbon particles in polymer matrix contributes to the formation of micro capacitances, which with interfacial polarization may be the cause of the improved dielectric properties. The use of composite filler allows us to avoid the agglomeration of ceramic and carbon particles and leads to the better dispersibility of the filler in the polymer matrix [35].

Similarly, with increasing KFTO(H)@CB filler content, the dielectric loss tangent in the region up to 10 kHz decreases significantly relative to that of PTFE/KFTO(H) composites without conductive filler (Figure 7c,d). For low-filled composites with 2.5 vol.%, the dielectric loss tangent does not change with the addition of carbon black. For composites with 5.0 and 7.5 vol.% of filler, the dielectric loss tangent and permittivity increase over the entire frequency range investigated. With the addition of iron-doped potassium titanate with carbon at 15 and 30 vol.%, the effect of optimization of dielectric properties is observed—i.e., the permittivity increases and the dielectric loss tangent decreases in the frequency range below 10 kHz. The dependencies detected point to a different effect of the carbon modification on the composites before and after the percolation threshold, which for this system is in the range 7.5–15 vol.%. Up to the percolation threshold, an increase in the filler fraction with the carbon additive is accompanied by a simultaneous increase in both permittivity and dielectric loss tangent. After the percolation threshold for samples with modification, the permittivity increase is more significant compared to a dielectric loss increase.

## 4. Conclusions

In this work, potassium titanate doped with iron ions K_1.6_Fe_1.6_Ti_6.4_O_16_ (KFTO(H)) characterized by a hollandite structure was obtained using the sol–gel method. KFTO(H) was modified with carbon black by the method of annealing in an argon atmosphere, resulting in powdered KFTO(H)@CB. PTFE/KFTO(H) and PTFE/KFTO(H)@CB composites with different contents of the solid-phase filler were prepared from the obtained powders by hot pressing. The dielectric properties of the composites in the frequency range 10^−1^ to 10^6^ Hz were investigated by impedance spectroscopy. The permittivity of PTFE/KFTO(H) composites with 30 vol.% of KFTO(H) content was 11 units and the tangent of dielectric losses was 0.23 at 1 kHz. The use of carbon-modified particles KFTO(H)@CB as a filler resulted in the increased porosity of the composites due to the reduced adhesion contact with the PTFE matrix. At the same time, the permittivity of PTFE/KFTO(H)@CB with 30 vol.% carbon-modified filler composite was 28 and the dielectric loss tangent was less than 0.1 at 1 kHz. The controlled location of CB in the ceramic–carbon interface could lead to optimized dielectric properties with a high ε and low tanδ in the polymer matrix composite based on PTFE. This goes against the current opinion that the enhancement in ε always comes with a high tanδ. Thus, the efficiency of three-phase composites with ceramic filler with carbon modification obtained by a simple annealing method in an inert atmosphere is shown.

## Data Availability

Not applicable.
